# *Melaleuca armillaris* Essential Oil in Combination With Rifaximin Against *Staphylococcus aureus* Isolated of Dairy Cows

**DOI:** 10.3389/fvets.2020.00344

**Published:** 2020-07-15

**Authors:** Daniel Buldain, Lihuel Gortari Castillo, Andrea Verónica Buchamer, Florencia Aliverti, Arnaldo Bandoni, Laura Marchetti, Nora Mestorino

**Affiliations:** ^1^Laboratorio de Estudios Farmacológicos y Toxicológicos (LEFyT), Facultad de Ciencias Veterinarias, Universidad Nacional de La Plata, La Plata, Argentina; ^2^Consejo Nacional de Investigaciones Científicas y Técnicas (CONICET), La Plata, Argentina; ^3^Facultad de Farmacia y Bioquímica, Cátedra de Farmacognosia, Universidad de Buenos Aires, Buenos Aires, Argentina; ^4^Instituto de Química y Metabolismo del Fármaco (IQUIMEFA), CONICET-Universidad de Buenos Aires, Buenos Aires, Argentina

**Keywords:** *Staphylococcus aureus*, dairy cows, *Melaleuca armillaris*, essential oil, rifaximin, checkerboard, synergism

## Abstract

*Staphylococcus aureus* is the major subclinical mastitis-causing pathogen in dairy cows. In some European and Latin American countries, rifaximin (RIF) is a commonly used therapy at drying off. Phytotherapeutics are alternatives for the treatment of infectious diseases. *Melaleuca armillaris* essential oil (EO) has been reported as a good antimicrobial against *S. aureus*. The aim of this work was to investigate, *in vitro*, the combined effect of EO and RIF to identify a synergic interaction against *S. aureus* in order to obtain enough information for subsequent pharmacokinetic/pharmacodynamic studies. The minimum inhibitory concentrations (MIC) for RIF, EO, and combinations of these against *S. aureus* strains were determined at pH 7.4, 6.5, and 5.0, representing intracellular conditions where *S. aureus* is usually located. The fractional inhibitory concentration index (FIC) and the index of antibacterial activity (*E*) were evaluated. The MIC of EO at pH 7.4 was 25–12.5 μL/mL and decreased with the acidity of the medium. RIF presented a high antimicrobial activity (0.032 μg/mL) against *S. aureus* regardless of the pH conditions. Combining RIF with EO, we found a synergic effect. A mix of 0.004 μg/mL of RIF and 12.5 μL/mL of EO led to a virtual eradication effect against wild-type strains at pH 7.4. Media acidification improves the EO/RIF activity, so EO would be a good adjuvant for RIF to treat staphylococcal infections and decrease antimicrobial resistance.

## Introduction

The growing threat of antimicrobial resistance to antibiotics has dramatically increased therapeutic failure and encourages exploring new therapeutic alternatives (1).

*Staphylococcus aureus* is one of the major contagious pathogens responsible for subclinical bovine mastitis. It is believed that the intracellular survival of this microorganism contributes to its recurrence (2). This microorganism is important for public health because of its virulence and the potential risk of genetic antimicrobial resistance determinants transference among animals, humans, and the environment (1).

Plant essential oils (EOs), complex mixtures of secondary metabolites, together with conventional antibacterials (ABs) may have synergistic effects (3). The different compounds present at the same time in plant extracts make it more difficult for the bacteria to develop simultaneous resistance mechanisms against each of them (4, 5).

*Melaleuca armillaris* (Sol. ex Gaertn.) Sm. is one of the most widely cultivated *Melaleuca* plants. Gas chromatography coupled with mass spectrometry research of *M. armillaris* EO revealed the presence of 1,8-cineol as the main component (6, 7). Inhibitory activity, *in vitro*, was found against several bacterial species, including *S. aureus* (8).

Difficulties in finding new AB molecules led to exploiting different strategies in those already in existence (9). Combined therapy, which associates conventional ABs with natural compounds such as EOs, represents a promising strategy to facing resistance to ABs (10). Synergistic combinations have greater efficacy and lower toxicity than their isolated components. There are some studies accounting for the synergistic activity between EOs and ABs (7, 11, 12) which suggest that the oil has potential to be used as an adjuvant in antimicrobial therapy.

Rifaximin (RIF) is an AB belonging to the ansamycin group, developed from rifamycin (13). It acts as an inhibitor of the RNA synthesis processes (14).

RIF has been traditionally applied in human medicine to treat travelers' diarrhea (15, 16) and hepatic encephalopathy (17). Valentin et al. (18) found rifampicin resistance in *Staphylococci*. Rifaximin and rifampicin are analogous. Consequently, RIF resistance in a microorganism can be compared to rifampicin resistance (19). Rifampicin resistance is mainly produced by chromosomal alteration of DNA-dependent RNA polymerase, which is the AB target (20, 21). There are reports evidencing a quick *in vitro* and *in vivo* resistance development to rifamycins when these are used as mono-drugs (22, 23). Therefore, it is usually combined with other compounds (20, 24).

Rifaximin usage in veterinary medicine is indicated for reproductive disorder treatments for cows and horses (25). Moreover, this antimicrobial is used as a good alternative to metronidazole to treat chronic enteropathy in dogs (26) as well as for localized treatment in bovine mastitis (27, 28). RIF usage in cows with mastitis has been approved by the European Agency for the Evaluation of Medicinal Products in Europe and is widely used in countries such as Italy, France, Poland, Austria, Estonia, and Israel. On the other hand, this antibiotic is also being widely used with the same purpose in South America, mainly in Argentina, Chile, and Uruguay. However, information about its pharmacodynamic and pharmacokinetic behavior is non-existent. There are only a few publications on its efficacy in milk cattle (29, 30).

There are no reports about RIF combined with essential oils. We previously found a synergic activity between *M. armillaris* essential oil and cloxacillin (7).

Thus, the aim of this study was to establish the *in vitro* antimicrobial activity of RIF combined with *M. armillaris* EO at different pH values (emulating intracellular conditions). This condition allows us to assess whether this EO is a good adjuvant for ansamycins in *S. aureus* treatment within the intracellular environment where it is usually located.

## Materials and Methods

### *M. armillaris* Essential Oil Extraction and Characterization

Leaf and herbaceous branch collection was carried out in Coronel Brandsen, Buenos Aires, Argentina (latitude 35°06′18.9″ S and longitude 58°10′57.0″ W). In July 2015, we obtained 44.85 kg of plant material. A sample portion was reserved for the identification and further storage at the LPAG herbarium at the Faculty of Agrarian and Forestry Sciences, UNLP (31). EO was obtained by steam distillation of the whole collected fresh biomass, yielding 550 ml of oil. Subsequently, the EO was dried with sodium sulfate anhydrous at room temperature, filtered using a cotton funnel, and stored in a glass amber bottle until use at 4°C.

The EO composition was analyzed before starting the antimicrobial assays with gas chromatography–flame ionization detection–mass spectrometry (GC-FID-MS), as we previously described (7), in order to avoid any variation in the EO composition during the storage until use.

### *S. aureus* Strains and Susceptibility Against RIF

Three wild-type (*n* = 3) *S. aureus*, isolated according to the National Mastitis Council procedure (32), from subclinical mastitis Holstein cows were used. The protocol was carried out following the Guide for the Care and Use of Agricultural Animals in Agricultural Research and Teaching (Federation of Animal Science Societies, FASS) and was approved by the Institutional Committee (CICUAL) of the Faculty of Veterinary Sciences, National University of La Plata (47.3.15J). For quality control, *S. aureus* ATCC 29213 was used. *S. aureus* susceptibility was checked with the disk diffusion test using rifampicin disk (5 μg) as a marker for the RIF susceptibility of *S. aureus* (33).

### Minimum Inhibitory and Bactericidal Concentrations of RIF and EO

The minimum inhibitory concentrations (MICs) of RIF and EO were determined by the broth microdilution method in 96-well polystyrene microplates. Mueller Hinton Broth (MHB) culture medium was used with the addition of 0.5% of Tween 80 to improve the EO dissolution. The MICs and minimum bactericidal concentrations (MBCs) were evaluated at three pH values (7.4, 6.5, and 5.0) in order to analyze what would happen at the intracellular level (inside cytosol or phagolysosome) where *S. aureus* is internalized. Hydrochloric acid 0.1 N for the broth pH adjustment was applied.

The antibiotic stock solution was prepared by using high-potency RIF (>99%, *w*/*w*; Santa Cruz Biotechnology, CA, USA). Methanol (Baker-Mallinckrodt, Phillipsburg, USA) was applied as the solvent and MHB as the diluent. The range of dilutions assessed was from 256 to 0.007 μg/mL using 2-fold serial dilution (34). In the case of the EO, a range of 50–0.1 μl/mL was tested. We applied the same procedure described by CLSI 2009 (34), with the addition of 0.5% Tween 80 for EO solubilization.

Each plate was inoculated with a final bacterial concentration of 5 × 10^5^ UFC/ml and incubated at 35°C for 18–24 h. MIC was established as the lowest concentration inhibiting bacterial growth. Positive and negative controls with MHB containing 0.5% Tween 80 were performed. Every determination for each strain was evaluated at the three different pH conditions by triplicate.

Once MIC was established, 25 μL was taken from each well-showing no evident bacterial growth. Then, these were inoculated individually in nutritive agar plates for colony counting after incubation at 35°C for 18–24 h. Thus, MBC was the first antimicrobial concentration in which the initial inoculum falls at 99.9%.

### Antimicrobial Activity of Combinations of RIF/EO

Once MIC determinations were established, a checkerboard technique (35) was carried out for the combinations of RIF and EO at pH of 7.4, 6.5, and 5.0. This led to establishing the presence or absence of a synergistic interaction between them against *S. aureus*. The microplate design comprised a 2-fold serial dilution column of RIF with a 2-fold serial dilution row of EO (both MIC controls). The additional wells contained RIF and EO in different proportions. The final volume was 200 μL, so it was added 100 μL of broth, 50 μL of the correspondent antibiotic concentration, and the same for the EO. The inocula were dispensed considering a final concentration of 5 × 10^5^ CFU/ml per well, as for the MIC determination explained above. Microplates were incubated at 35°C for 18–24 h, and every determination was carried out in triplicate. The results were interpreted by considering the fractional inhibitory concentration indexes (FICs), where this index is defined as the sum of the quotients between the MIC of the single and the combined antimicrobials. In this way, synergism exists if FIC ≤ 0.5, partial or low synergism (PS) if 0.5 < FIC < 1, indifference or addition (I) if 1 ≤ FIC < 2, and antagonism (A) if FIC ≥ 2 (35).

### Antibacterial Activity Index of Rifaximin Alone and Combined With EO

Once the MICs of EO, RIF, and their combinations were identified, the data were used to perform time-kill assays so as to evaluate the antibacterial activity index (*E*). Each *S. aureus* strain was exposed to different concentrations (0.5, 1, 2, 4, and 8 MICs) of RIF and EO/RIF combinations. In the last case, the mixture which had the lowest FIC value was established as the MIC and the proportions of both components were retained. For quality control, *S. aureus* ATCC 29213 was used.

Considering the positive (without antimicrobials) and negative (without antimicrobials and inocula) controls, seven tubes were prepared. Each one contained a final volume of 1 ml including MHB with 0.5% Tween 80 (pH 7.4, 6.5, and 5.0), antimicrobials, and a final inoculum of 5 × 10^5^ colony forming units (CFU)/ml, depending on the case. Incubations were carried out at 35°C. Time-kill curves by bacterial plate count at the initial time, 2, 4, 8, 12, and 24 h incubating at 35°C by 24 h were performed. This assay was carried out in triplicate for each strain.

Thereafter, the results were applied into a CFU/ml *vs*. time graph design to evaluate the antibacterial activity index (*E*). Once *E* is assessed, an index *vs*. antibiotic concentration (Log_10_) graph is generated to compare any effect with the presence of EO. The wild strains were grouped by obtaining an *n* = 3 (using the mean of triplicates for each strain). Graphics were plotted using the GraphPad Prism 6 program.

The *E* was quantified as the difference between the Log_10_ values of the number of viable bacteria (in colony forming units per milliliter) at the initial time (nt-0) and at the end of the test (nt-24). This is represented in the following equation: *E* = nt-24 – nt-0. Three theoretical breakpoints were applied to assess *E*: a) bacteriostatic effect, *E* = 0 (there are no changes in the value of nt-0); b) bactericidal effect, *E* = −3 (there is a reduction of ≥3 Log10 of nt-0; and c) effect of virtual eradication of bacteria, *E* = −4 (there is a reduction of ≥4 Log10 (99.99%) with respect to the Log of nt-0 (36).

## Results

**Figure 1:**
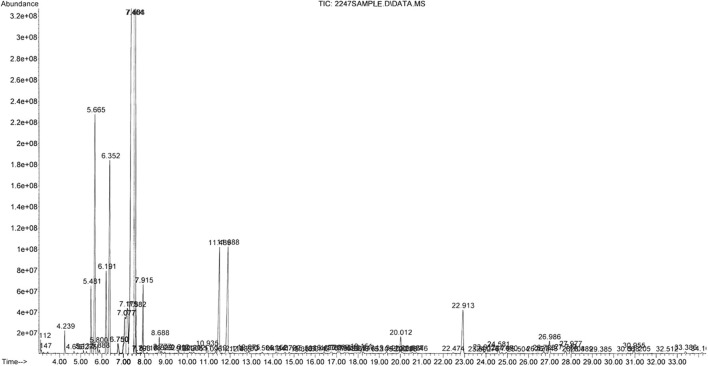
Gas chromatographic profile of the *Melaleuca armillaris* essential oil used (7).

After EO extraction, we obtained 550 ml of this, which resulted in a yield of 1.22%, *v*/*w* (volume/100 g of fresh material). Table 1 summarizes the compounds found by chromatographic analysis of the EO (Figure 1 shows the gas chromatogram).

**Table 1:** Relative percentage composition of *Melaleuca armillaris* essential oil (EO).

**Compounds**	**Area (%)**	**Compounds**	**Area (%)**
1,8-Cineole	72.3	β-Caryophyllene	0.5
Limonene	7.8	α-Terpinene	0.2
α-Pinene	6.0	*trans*-β-Ocimene	0.2
Myrcene	2.2	Geranyl acetate	0.2
β-Pinene	2.2	α-Phellandrene	0.1
α-Thujene	1.5	Terpinolene	0.1
p-Cymene	1.4	δ-Terpineol	0.1
Terpinen-4-ol	1.4	Aromandendrene	0.1
α-Terpineol	1.4	Geranyl isobutyrate	0.1
Sabinene	1.0	*cis*-Calamenene	0.1
γ-Terpinene	0.5	Oxi-Caryophyllene	0.1

Three wild-type strains (*n* = 3) and a reference strain (ATCC 29213) of *S. aureus* were tested against RIF, EO, and their combinations at pH 7.4, 6.5, and 5.0. The MICs obtained and the FICs in each pH condition are listed in Table 2. The MBC obtained for each strain and the pH condition tested for EO and RIF are shown in Table 3, including the relation MBC/MIC.

**Table 2:** Fractional inhibitory concentration indexes obtained for the EO/RIF combination under different pH conditions *vs*. individual MICs.

**Strains**	**pH 7.4**				**pH 6.5**				**pH 5.0**			
	**MIC** **EO** **(μL/mL)**	**MIC** **RIF** **(μg/mL)**	**MIC** **EO/RIF** **(μL/mL)/(μg/mL)**	**FIC**	**MIC** **EO** **(μL/mL)**	**MIC** **RIF** **(μg/mL)**	**MIC** **EO/RIF** **(μL/mL)/(μg/mL)**	**FIC**	**MIC** **EO** **(μL/mL)**	**MIC** **RIF** **(μg/mL)**	**MIC** **EO/RIF** **(μL/mL)/(μg/mL)**	**FIC**
ATCC 29213	25	0.032	12.5/0.002	0.56	25	0.032	6.25/0.004	0.37	12.5	0.032	3.1/0.004	0.38
SA 13	12.5	0.032	6.25/0.002	0.56	12.5	0.032	3.1/0.008	0.50	6.25	0.032	1.6/0.004	0.38
SA 96	12.5	0.032	6.25/0.002	0.56	12.5	0.032	3.1/0.008	0.50	6.25	0.032	1.6/0.004	0.38
SA 139	12.5	0.032	6.25/0.002	0.56	12.5	0.032	3.1/0.008	0.50	6.25	0.032	1.6/0.004	0.38

*MIC, minimum inhibitory concentration; EO, essential oil; RIF, rifaximin; FIC, fractional inhibitory concentration*.

**Table 3:** Minimum bactericidal concentrations (MBC) of EO and RIF at pH 7.4, 6.5, and 5.0 and the relation MBC/MIC in the three conditions.

**Strains**	**pH 7.4**				**pH 6.5**				**pH 5.0**			
	**MBC** **EO** **(μL/mL)**	**MBC/MIC** **EO**	**MBC** **RIF** **(μg/mL)**	**MBC/MIC** **RIF**	**MBC** **EO** **(μL/mL)**	**MBC/MIC** **EO**	**MBC** **RIF** **(μg/mL)**	**MBC/MIC** **RIF**	**MBC** **EO** **(μL/mL)**	**MBC/MIC** **EO**	**MBC** **RIF** **(μg/mL)**	**MBC/MIC** **RIF**
ATCC 29213	50	2	0.512	16	50	2	0.512	16	25	2	0.064	2
SA 13	25	2	0.512	16	25	2	0.512	16	25	4	0.128	4
SA 96	50	4	0.512	16	25	2	0.512	16	12.5	2	0.128	4
SA 139	25	2	0.512	16	25	2	0.512	16	12.5	2	0.128	4

*MIC, minimum inhibitory concentration; EO, essential oil; RIF, rifaximin; MBC, minimum bactericidal concentrations*.

**Figure 2:**
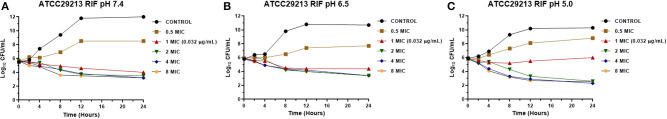
Time-kill curve for rifaximin on *Staphylococcus aureus* ATCC 29213 at pH 7.4 **(A)**, 6.5 **(B)**, and 5.0 **(C)** [minimum inhibitory concentration (MIC) = 0.032 μg/mL].

**Figure 3:**
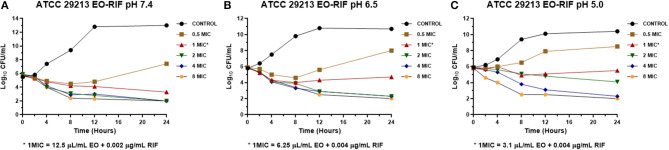
Time-kill curve for combinations of essential oil/rifaximin on *Staphylococcus aureus* ATCC 29213 at pH 7.4 **(A)**, 6.5 **(B)**, and 5.0 **(C)** [minimum inhibitory concentration (MIC) = 0.032 μg/mL].

**Figure 4:**
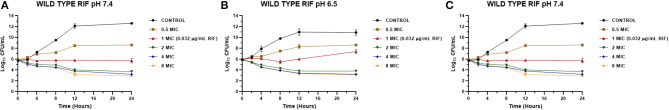
Time-kill curve for rifaximin on *Staphylococcus aureus* wild types (*n* = 3, using the mean of triplicates for each strain) at pH 7.4 **(A)**, 6.5 **(B)**, and 5.0 **(C)** [minimum inhibitory concentration (MIC) = 0.032 μg/mL].

**Figure 5:**
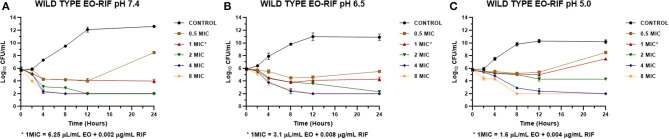
Time-kill curve for combinations of essential oil/rifaximin on *Staphylococcus aureus* wild types (*n* = 3, using the mean of triplicates for each strain) at pH 7.4 **(A)**, 6.5 **(B)**, and 5.0 **(C)** [minimum inhibitory concentration (MIC) = 0.032 μg/mL].

The MICs of EO and RIF, shown in Table 2, were used to perform time-kill assays at pH 7.4, 6.5, and 5.0. In Figures 2, 4, it is possible to observe the effect of the different RIF concentrations against the *S. aureus* strains (reference and wild type, respectively). The acidity seems to influence the bactericidal activity. At pH 5.0, concentrations of 4 and 8 MIC of RIF had a stronger antimicrobial effect after 24 h with respect to the situation at pH 7.4. The addition of EO improves the antimicrobial activity of RIF, requiring less concentration of it for the same effect against the reference (Figure 3) and wild-type strains (Figure 5).

**Figure 6:**
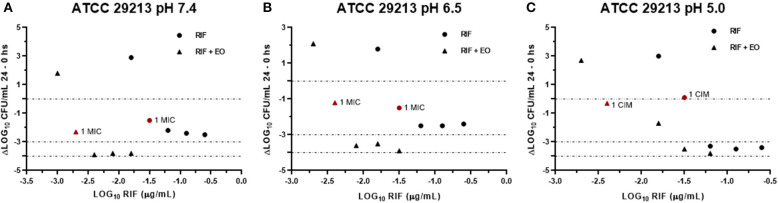
Graphic representation of the antibacterial effect (*E*: ΔLog CFU/ml 24–0 h) of rifaximin against *Staphylococcus aureus* ATCC 29213 at pH 7.4 **(A)**, 6.5 **(B)**, and 5.0 **(C)** in the presence and absence of EO.

**Figure 7:**
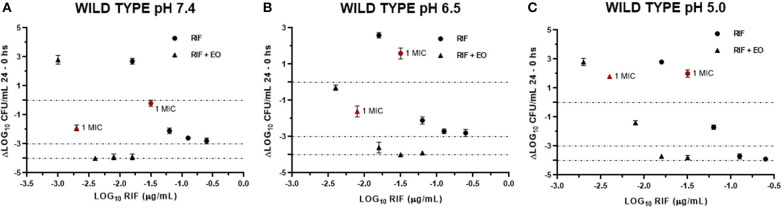
Graphic representation of the antibacterial effect (*E*: ΔLog CFU/ml 24–0 h) of rifaximin against *Staphylococcus aureus* wild type (*n* = 3, using the mean of triplicates for each strain) at pH 7.4 **(A)**, 6.5 **(B)**, and 5.0 **(C)** in the presence and absence of EO.

Data obtained from the time-kill assays were used to establish the antibacterial activity index (*E*). Figures 6, 7 show the E *vs*. Log_10_ (RIF concentration) and highlight again the synergic antibacterial effect obtained by the addition of EO to the culture with RIF against *S. aureus* (reference and wild-type strains, respectively). In this way, it is possible to observe that the amount of RIF necessary to decrease the bacterial inoculum in 24 h is lower in the presence of EO.

## Discussion

### EO Extraction and Characterization

Chromatographic analysis of the EO revealed the presence of 1,8-cineol as the main component (72.3%) and, in lesser magnitude, limonene (7.8%) and α-pinene (6.0%). These chemical compounds are commonly present in EOs with high antimicrobial activity, particularly 1,8-cineol. This compound is a cyclic monoterpene and is, in general, the main component in the essential oil of *Eucalyptus* species (37). In several studies, 1,8-cineol has also been reported as the main component of the EO of *M. armillaris*: 33.9% in Egypt, 85.8% in Tunisia, and 80.2% in Brazil (38–40). However, these results do not agree with other authors such as Amri et al. (8) and Siddique et al. (41), who determined markedly low concentrations of 1,8-cineol: 3.6% and 0.29%, respectively. Two other major compounds present in the EO with high antimicrobial activity are limonene and α-pinene (42). These differences are common in aromatic plants and can be attributed both to genetic varieties and/or exogenous variables. For these reasons, it is essential that the chemical composition of the EO we used in this study is shown.

Standardization of the EO composition is very important for pharmaceutical use. Obtaining clones with their own specific characteristics would be a probable solution to this issue. This would facilitate the EO to be used for the establishment of commercial crops. In this way, the problem of heterogeneity would be obviated, resulting in the production of homogeneous EO quality required by market standards. Siani et al. (43) carried out a vegetative propagation of one chemotype of *Lippia alba* cultivated in six different locations in Brazil under several different environmental conditions. After EO extraction, only slight variations of the content and qualitative profiles of the compounds were observed.

On the other hand, it would be possible to obtain some of the compounds available in the market in case it is necessary to enrich the obtained EO. This will allow assessing the quality and efficacy. However, it is necessary to understand which variation on the composition has an effect on the biological activity to establish a range of variations without difference.

### Inhibitory and Bactericidal Activity of EO

The MIC of *M. armillaris* EO against *S. aureus* ATCC 29213 was 25 μL/mL at pH 7.4 and 6.5, while at pH 5.0 it decreased by half. This same pattern of antimicrobial activity was observed in all the wild-type strains tested (SA13, SA96, and SA139); the MIC was 12.5 μL/mL at pH 7.4 and 6.5, decreasing to 6.25 μL/mL when the pH of the medium was acidified. Something similar occurred when evaluating the MBC. This parameter decreased between two and four times at pH 5.0 with respect to pH 7.4 (Table 3).

In addition, the high content of 1,8-cineol may be one of the factors contributing to the antibacterial activity of the EO. This compound has been attributed with the permeabilization of the membranes of microorganisms (such as *S. aureus*) as an antimicrobial action due to its high hydrophobicity (44, 45). Yañez Rueda and Cuadro Mogollón (46) found an important antibacterial activity of the *Eucalyptus globulus* essential oil against *S. aureus* ATCC 29213. The MIC was 12.4 μg/mL. It is noteworthy that the composition of the *E. globulus* EO was similar to that of the *M. armillaris* evaluated in this study: with 1,8-cineol (82.3%), followed by limonene (3.7%), α-pinene (3.2%), terpinen-4-ol (1.4%), α-terpineol (1.2%), β-myrcene (1.12%), and α-terpinene (1.1%), among others. Thus, it is likely that a synergism between these components is particularly effective against *S. aureus* strains.

The mechanism of the antimicrobial action of the *M. armillaris* EO against *S. aureus* has not yet been investigated. Hayouni et al. (47) studied the antimicrobial activity of this plant's oil against different species of *Lactobacillus*. As 1,8-cineol was the main component found in a concentration of 68.9%, these authors suggested as a hypothesis that this compound could have destabilized the bacterial cytoplasmic membrane, as was demonstrated by Li et al. (48). However, the postulated mode of action of *M. armillaris* by Hayouni et al. (47) also involves a minority of the components found (α-pinene, terpinen-4-ol, sabinene, β-myrcene, and α-terpinene, among others). According to these authors, these molecules interact with the cell membrane, where they are dissolved in the phospholipid bilayer and aligned between the chains of fatty acids. The distortion of the physical structure would cause the expansion and destabilization of the membrane, increasing its fluidity, which would increase the passive permeability.

A compound is bacteriostatic if the MBC/MIC ratio is >4 (49). The capacity of an EO to act as a bactericidal or bacteriostatic will depend on the chemical composition of this extract. When analyzing the MIC and MBC of the EO of *M. armillaris* against strains SA13, SA96, and SA139, we found that these parameters were very close, with a ratio of MBC/MIC ranging between 2 and 4 (Table 3). Therefore, considering the above-mentioned definition, the EO would act as a bactericidal antimicrobial maintained with different pH values.

### Inhibitory and Bactericidal Activity of RIF

The rifaximin MIC values for quality control to establish the susceptibility of *S. aureus* are not included in the CLSI documents, so we compared our results with the information published for rifampicin in the CLSI document VET01-S2 (CLSI 2013), which establishes a range of 0.004–0.016 μg/mL (it is the only ansamycin that appears in the document).

A MIC of 0.032 μg/mL was obtained for all the studied strains (reference and wild-type strains). This value is higher than the value established for rifampicin (0.004–0.016 μg/mL) by CLSI 2013 (33). Broth acidification did not produce a variation in the inhibitory activity of RIF; the MIC was the same at the three pH values evaluated.

In this study, we observed that RIF had high antimicrobial activity against the *S. aureus* strains ATCC 29213, SA13, SA96, and SA139. The obtained MIC values were similar for all strains analyzed (0.032 μg/mL). This indicates an important antibiotic potency against *S. aureus*. Moreover, Hoover et al. (50) found values ranging between ≤0.015 and 0.03 μg/mL for strains of these types of species, coinciding with our results. In another study carried out by Pistiki et al. (19), the reported MIC values of RIF were ≤0.25 μg/mL. No changes were observed in the MIC values at different pH levels. However, MBC showed differences when the antimicrobial activity was analyzed at the most acidic pH level. The MBC was 0.512 μg/mL for all strains at pH 7.4 and 6.5, whereas at pH 5.0 it decreased to 0.064 μg/mL for strain ATCC 29213 and to 0.128 μg/mL for the three wild-type strains. The MBC/MIC for all the analyzed strains was 16 at pH 7.4 and 6.5, working as a bacteriostatic antimicrobial. But at pH 5.0, the ratio ranged between 2 and 4 (Table 3), meaning that the antimicrobial activity is bactericidal at a lower pH level.

When we evaluated with the time-kill assay how the presence of RIF affects the growth of the reference strain of *S. aureus*, it was observed that it grew at a concentration of 0.5 MIC (0.016 μg/mL) at the three pH conditions, which was always lower than the control growth curve (Figure 2). Concentrations of 2, 4, and 8 MIC showed curves with similar profiles, independently of the broth pH. The concentrations evaluated (0.016–0.256 μg/mL) allowed us to obtain a decrease of at least 3 Log_10_ of the initial inoculum at pH 7.4 and 6.5. In contrast, at pH 5.0, the bactericidal effect was achieved for 2, 4, and 8 MIC; this coincides with the results observed in the determination of MBC since the values obtained for this parameter were 0.512 μg/mL (pH 7.4 and 6.5) and 0.064 μg/mL (pH 5.0).

Something similar was observed for the wild-type strains SA13, SA96, and 139 (Figure 4) because bactericidal effects were not seen with the concentrations evaluated at pH 7.4 and 6.5. At pH 5.0, the initial inoculum decreased more than 3 Log_10_ with concentrations of 4 and 8 MIC, corresponding to concentrations of 0.128 and 0.256 μg/mL, respectively. These values were also consistent with those obtained in the determination of MBC since, for these strains and at this pH, this value was 0.128 μg/mL.

It is possible to observe a reduction in the bacterial count of *S. aureus* strains against RIF as early as 2 h after starting the time-kill assay. For this antibiotic, it is not yet well-established whether it is an antimicrobial time- or a concentration-dependent one. However, some studies report that rifampin (belonging to the ansamycin family) acts as a concentration-dependent antimicrobial bactericide (51, 52). On the other hand, this antibiotic should be combined with other antimicrobials, such as penicillins resistant to penicillinase, vancomycin, or trimethoprim, due to it quickly selecting resistant strains when used as a mono-drug. Resistance to rifampin takes place by chromosomal mutation and develops easily in most bacteria. Such mutants show stable changes in the RNA polymerase (the target site of the antimicrobial action), preventing fixation. These result from mutations in the *rpoB* gene, which codes for the sub-subunit of the bacterial RNA polymers, where these antibiotics bind (53).

### Antimicrobial Activity of EO/RIF Combinations

In the literature, there are no reports on the combinations of ansamycins with essential oils against *S. aureus*. There are only a few reports of natural extracts with this type of drugs. For example, Liu et al. (54) found a strong synergistic activity between rifampicin and manuka honey against strains of *S. aureus*-producing biofilm.

Combining RIF with the EO of *M. armillaris*, a synergistic effect was found, and the antibiotic activity was enhanced, particularly at pH 6.5 and 5.0, in all the strains assayed. At pH 7.4, there were combinations obtained that presented partial synergism, with FIC = 0.56, very close to the FIC value considered for synergism (0.5). In this case, it was possible to decrease, for the four strains studied, 16 times the MIC of RIF with a decrease to half of the MIC of the EO with respect to each compound applied alone. At pH 6.5, the decrease in the inhibitory concentration of the antibiotic was lower (four times for the wild-type strains and eight times for the reference strain). However, the drop in the amount of EO necessary to enhance the antibiotic antimicrobial activity was more evident here since, for all the strains, the decrease was four times the MIC of the plant extract. Finally, at pH 5.0, the MIC of RIF decreased eight times for all strains. In the last case, the decrease in the inhibitory concentration of the EO is much more evident since it decreased by a factor of 4 again. Therefore, as a result of the significant reduction in the inhibitory concentrations at pH 5.0, both essential oil and antibiotic, the FIC values obtained were of 0.38, which show an important synergy between them.

Thus, RIF has a potentiated effect by the sum of two factors: combination with the essential oil and the acidification of the culture medium.

In the time-kill assay, we could observe a slow decrease in the bacterial count for RIF alone that occurs mainly between 12 and 24 h after starting the experiment. On the other hand, when it was combined with the EO of *M. armillaris*, a strong drop in the bacterial count was observed at 4 h, with a maximum decrease at 8 h (Figures 3, 5). Moreover, it is evident that the drop of the slopes of the curves is more important in the presence of the mixture compared with the EO alone since this produces the greatest decrease in the viable cell counts at 12 h. Xiao et al. (55) combined *Origanum vulgare* essential oil with rifampin, managing to eliminate *S*. *aureus* strains in a stationary phase in 24 h. This effect is different when both compounds act alone, thus could not kill all the inocula at the same time. Therefore, combining some essential oils with ansamycins seems to be useful in treating staphylococcal infections.

In another respect, by analyzing bacteria inoculum decrease with *E* (Figures 6, 7), it is possible to graphically observe how *S. aureu*s is inhibited in a higher magnitude with lower RIF concentrations when *M. armillaris* EO is present. This means that the EO allows a reduction of the amount of necessary antibiotic for microorganism inhibition. At pH 7.4 and 6.5, RIF was not able to produce any bactericidal effect on its own at the tested concentrations, unlike as occurred at pH 5.0, while when combined with the EO of *M. armillaris* allowed achieving, with lower concentrations, bactericidal effects even close to virtual eradication. For example, a mixture of 0.004 μg/mL RIF and 12.5 μL/mL EO allowed achieving a virtual eradication effect against the wild-type strains at pH 7.4 (Figure 7). Something similar happened with the reference strain (Figure 6). The acidification of the media improves the activity of the essential oil/rifaximin combination. These outcomes are similar to those previously obtained by combining cloxacillin with EO (7). In both cases, the acidic conditions increased the synergic effect and the bactericidal activity, which is interesting if we are dealing with intracellular infections.

There are a few studies *in vivo* with EO; however, Byung-Wook et al. (56) treated cow's clinical mastitis with essential oil of *O. vulgare*. A decrease in infection by *S. aureus* without causing swelling, redness, pain, and heat to the udder has been observed. There are some essential oil-based products for intramammary application in the market, such as Phyto-Mast. This is recommended for intramammary use in lactation and dry-off. Thymus EO is the antimicrobial active component (57), and its residues (main component) were only detected 12-h post-treatment in milk using goats and cows as animal models. The activity did not present any irritating and inflammatory effects (58, 59). These findings allow us to consider the feasibility of administering the essential oil of *M. armillaris* intramammary in the future.

## Conclusions

We observed a reduction of the necessary antibiotic concentration needed to inhibit *S. aureus* by combining *M. armillaris* essential oil with rifaximin even at different pH conditions. The *in vitro* bactericidal activity of the mixture was very important since, with the RIF/EO mixture, an effect close to a virtual eradication was obtained.

As was previously mentioned, rifaximin is highly used in humans and animals and contributes to antimicrobial resistance. It is known that when rifamycins are used alone, they select quickly antimicrobial resistance *in vivo* and *in vitro*, so it is required to use them in combination with other compounds to avoid that effect.

Consequently, we conclude that the essential oil of *M. armillaris* could be considered as an effective adjuvant for therapies with RIF. Nonetheless, it is essential to assess security by using cell and animal models.

## Data Availability Statement

The raw data supporting the conclusions of this article will be made available by the authors, without undue reservation.

## Ethics Statement

*S. aureus* isolations used in this work belong to the microorganism's collection of our laboratory obtained in previously projects. The protocol of bacterial isolation was carried out following the Guide for the Care and Use of Agricultural Animals in Agricultural Research and Teaching (Federation of Animal Science Societies – FASS) and was approved by the CICUAL of the Faculty of Veterinary Sciences, National University of La Plata (47.3.15J).

## Author Contributions

NM conceived and designed the experiments. DB: performed all the experimental assay, statistical analysis, and wrote the manuscript. LC, AVB, FA, and LM contributed to the experimental assays. AB performed the EO quality assay. All authors contributed to the redaction and approved the final manuscript.

## Conflict of Interest

The authors declare that the research was conducted in the absence of any commercial or financial relationships that could be construed as a potential conflict of interest.
